# Supplementary material for: Targeted Treatment Options of Recurrent Radioactive Iodine Refractory Hürthle Cell Cancer
Source: Cancers (Basel). 2019 Aug 15;11(8):1185. doi: 10.3390/cancers11081185 (PMC6721552; doi:10.3390/cancers11081185)
Supplement: Supplementary file 1 [file cancers-11-01185-s001.pdf]

Supplementary material

Targeted Treatment Options of Recurrent Radioactive Iodine Refractory Hürthle Cell Cancer

Mehtap Derya Aydemirli, Willem Corver, Ruben Beuk, Paul Roepman, Nienke Solleveld-Westerink, Tom van Wezel, Ellen Kapiteijn and Hans Morreau

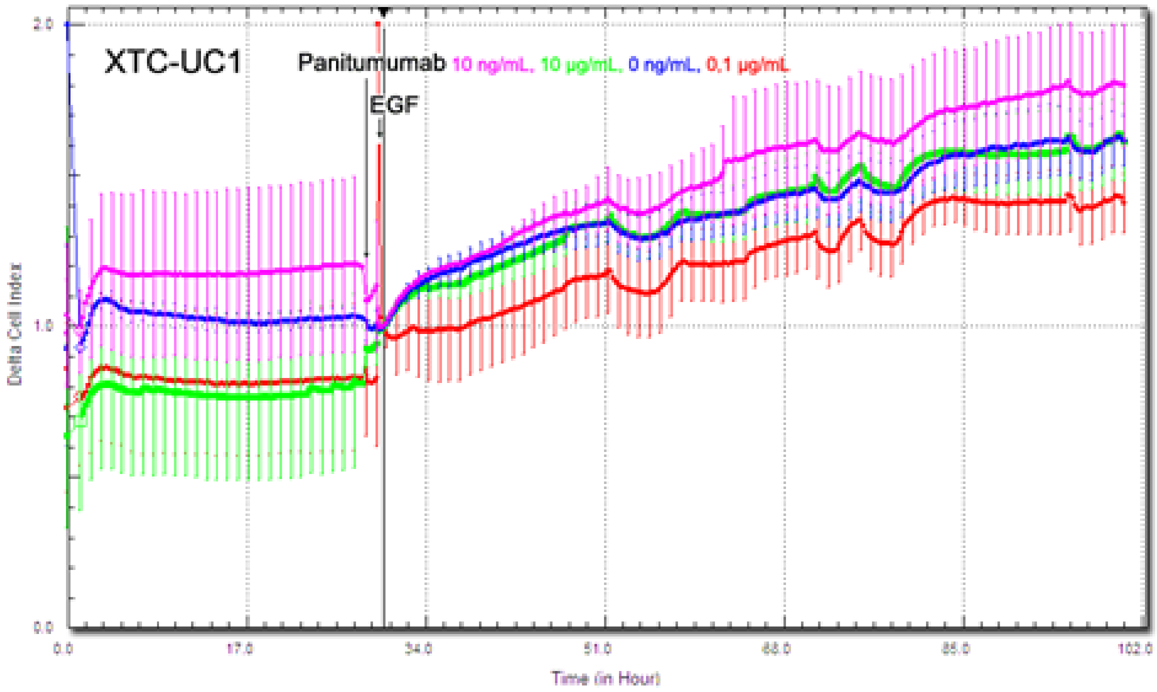

Figure S1. Assessment of cell proliferation effects of panitumumab, using impedance-based real time detection of cellular viability with xCELLigence system Real Time Cell Analyzer RTCA-MP, on XTC.UC1. After serum starvation for 28 h, cells were incubated with 0, 10, 100 and 10,000 ng/mL panitumumab, followed by EGF stimulation an h later. XTC-UC1 cells showed a substantial increase in impedance upon EGF stimulation. However, compared to cells pretreated with panitumumab, no difference in impedance was observed. X-axis: time expressed in h; Y-axis: ΔCell Index normalized for timepoint when EGF was added.

C33A

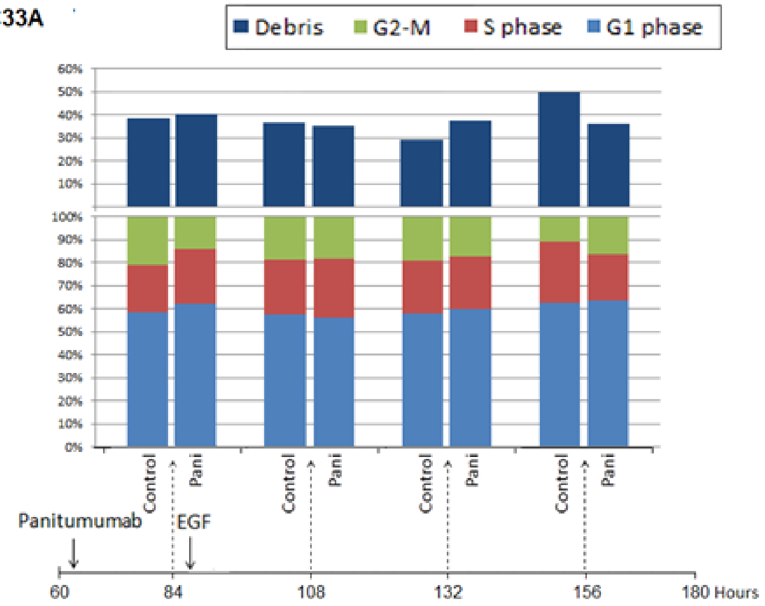

FTC-236

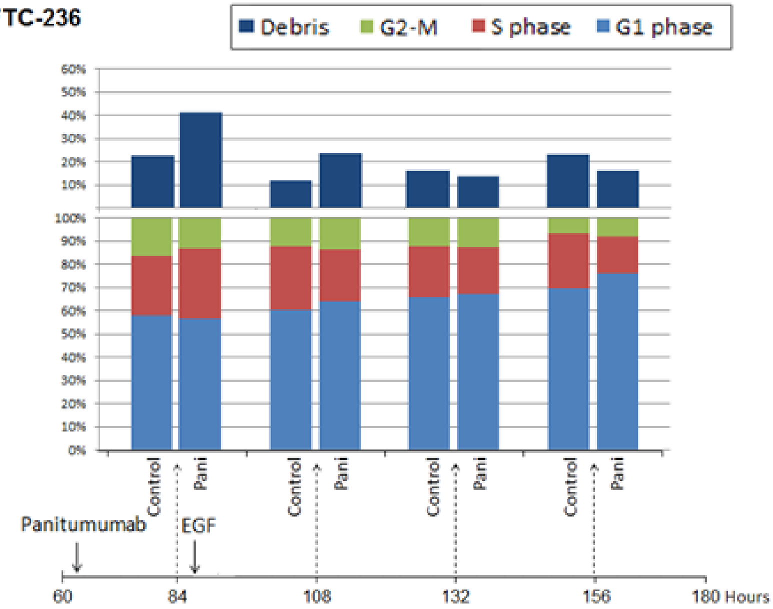

BHP 2-7

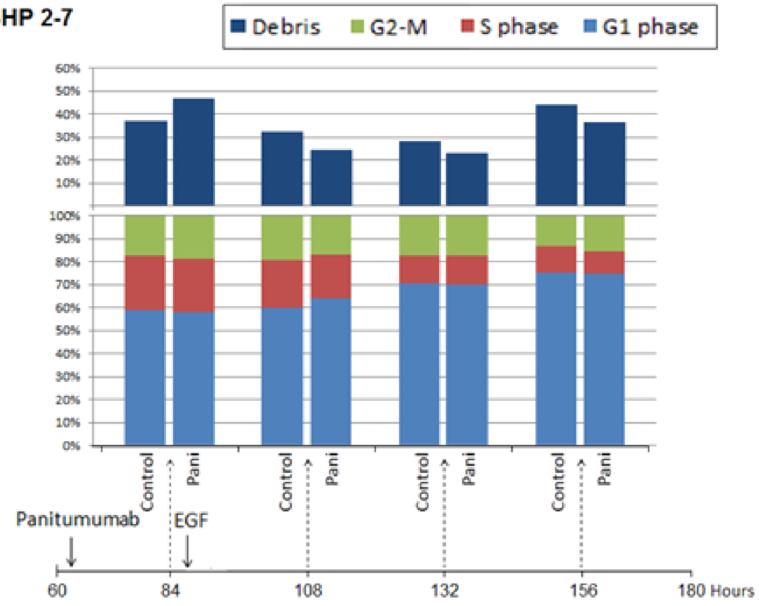

Figure S2. Flow cytometric analysis of cell cycle distribution. Cells were serum starved (60 h), treated with panitumumab (24 h) and EGF stimulated (72 h). Cell line C33A (used as negative control for the lack of EGFR) showed no response to EGF stimulation and panitumumab treatment resulted in a similar cell cycle distribution as untreated cells. BHP 2-7 cells showed S-phase reduction after 72 h of EGF incubation, likely due to reaching confluency because of cell proliferation. Panitumumab treatment had no effect on cell cycle distribution in BHP 2-7. FTC-236 had an increased G1 when EGF stimulated, suggestive of proliferation. No substantial effect of panitumumab on cell cycle distribution and debris was observed. In the upper graph the percentage of debris is shown out of the total events. In the lower part G0-G1, S and G2-M phases are shown as a percentage of the total number of cells.

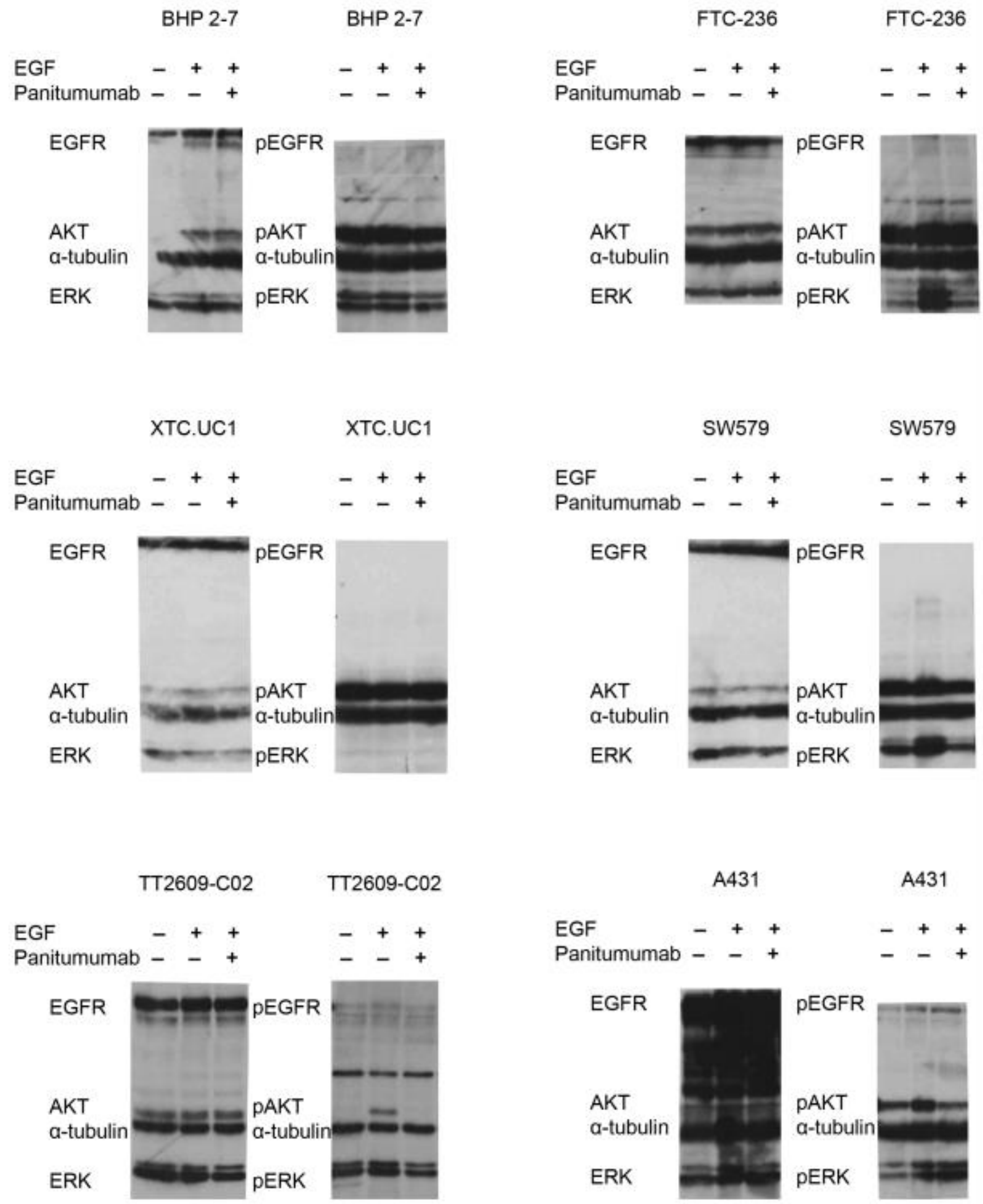

Figure S3. Additional exploratory western blot assessments, electrochemiluminescence (ECL). Thyroid cancer cell lines (BHP 2-7, FTC-236, XTC.UC1, SW579, TT2609-C02) and control (A431) were serum-starved, or treated with(out) panitumumab and with EGF stimulation. The presence of the proteins of EGFR, AKT and ERK was confirmed by western blotting and phosphorylation was assessed (pEGFR, pAKT, pERK), loading control was α-tubulin. Unfortunately, pEGFR expression is visualized suboptimally (lane 2). In BHP 2-7 and TT2609-C02, pERK shows constitutive activation, similar to Figure 5. Comparatively, in FTC-236, SW579, pERK shows higher intensity upon EGF stimulation (lane 2), that may be lowered back in FTC-236 and SW579 to control level (lane 1) upon

panitumumab treatment (lane 3). In control cells A431, pERK levels remained high despite panitumumab. Unfortunately, in XTC.UC1, the expression of pERK was visualized suboptimally; however, the western blot for ERK and pERK expression was repeated for XTC.UC1 (not shown here), yielding similar results as in Figure 5. In TC cell lines BHP 2-7, SW579, XTC.UC1 and FTC-236, pAKT showed constitutive activation. Higher intensities in the latter two cell lines are not be determined reliably based on these blots in comparison to other cell lines, due to distinct blots. In TT2609-C02, pAKT expression was low in general, but could be lowered to control level with panitumumab. The latter also applied to control cells A431, showing no constitutive pAKT.

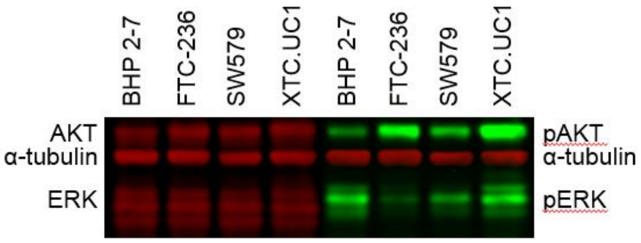

Figure S4. Additional western blot assessments (immunofluorescent). Untreated thyroid cancer cells (BHP 2-7, FTC-236, SW579, XTC.UC1) were lysated and loaded onto the same gel. Western blot analysis confirmed the presence of AKT, ERK (both red-fluorescent) in all cells and α-tubulin (red-fluorescent) as loading control, see the four consecutive lanes on the left. Expression of pAKT (green-fluorescent) and pERK (green-fluorescent) is shown on the four lanes on the right. Similar to the western blot analysis in Figure 5, pAKT showed higher intensities in the NHG-harboring FTC-236 and XTC.UC1 cells as compared to BHP 2-7 and SW579. pERK showed higher intensities in BHP 2-7 cells and XTC.UC1 as compared to FTC-236 and SW579.

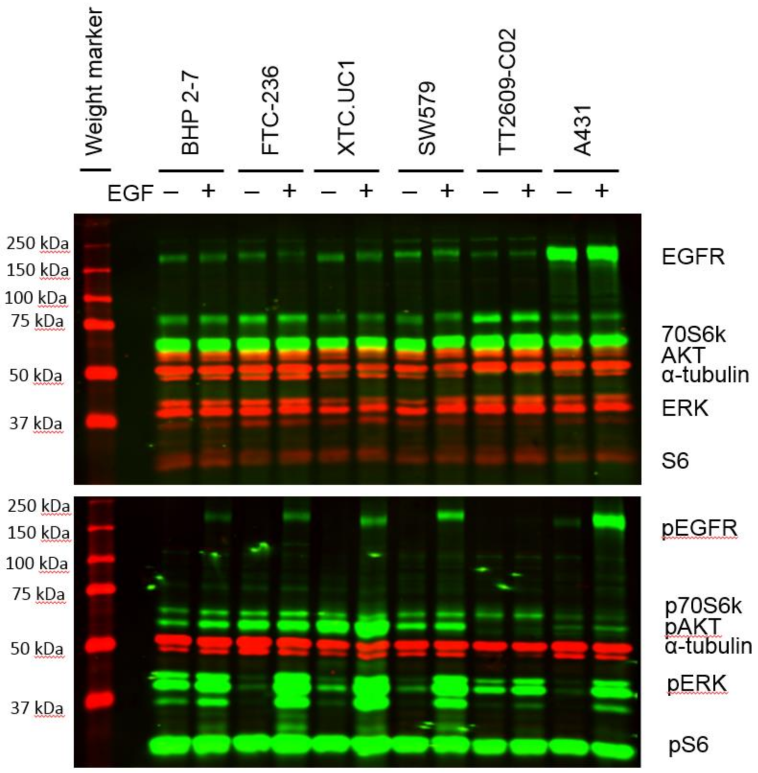

Figure S5. Additional western blot assessments (immunofluorescent). EGF unstimulated (–) or stimulated (+) thyroid cancer cells (BHP 2-7, FTC-236, XTC.UC1, SW579, TT2609-C02) and EGFR overexpressing control A431 were lysated and loaded onto two gels prepared from the same mixture, electrophoresis, blotted and processed simultaneously. Western blot analysis confirmed the presence of EGFR, 70S6k (#2708, CST) (both green-fluorescent) and AKT, ERK, S6 (#2317, CST) (red-fluorescent) in all cells and α-tubulin (red-fluorescent) as loading control, see the upper blot.

Expression of pEGFR, p70S6k (#9234, CST), pAKT, pERK, pS6 (#2211, CST) (all green-fluorescent) is shown on the lower blot. Similar to the western blot analysis in Figure 5, pAKT showed constitutive activation with higher intensities in the NHG-harboring FTC-236 and XTC.UC1 cells as compared to BHP 2-7, SW579, TT2609-C02, A431. Similar to Figure 5, pERK showed constitutive activation with higher intensities in unstimulated BHP 2-7 and TT2609-C02 cells as compared to the other cells. Similar to Supplemental figure 2, XTC.UC1 cells show baseline pERK, however upon EGF stimulation this signal is highly intensified and appears not constitutively activated in contrast to the BHP 2-7 or TT2609-C02 cells. p70S6k and pS6 (both downstream effectors of mTOR) appear similarly expressed across all thyroid cells.

A.

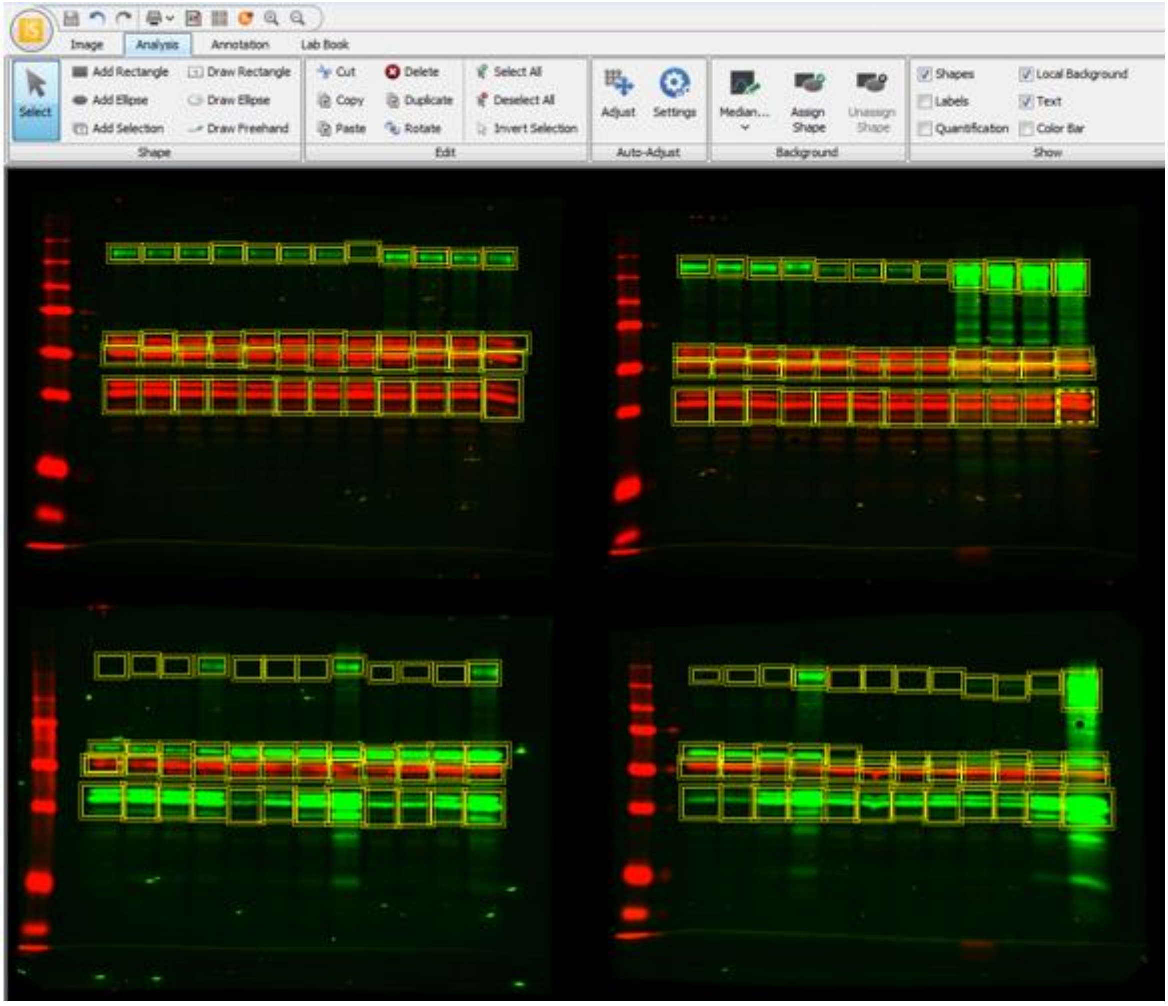

B.

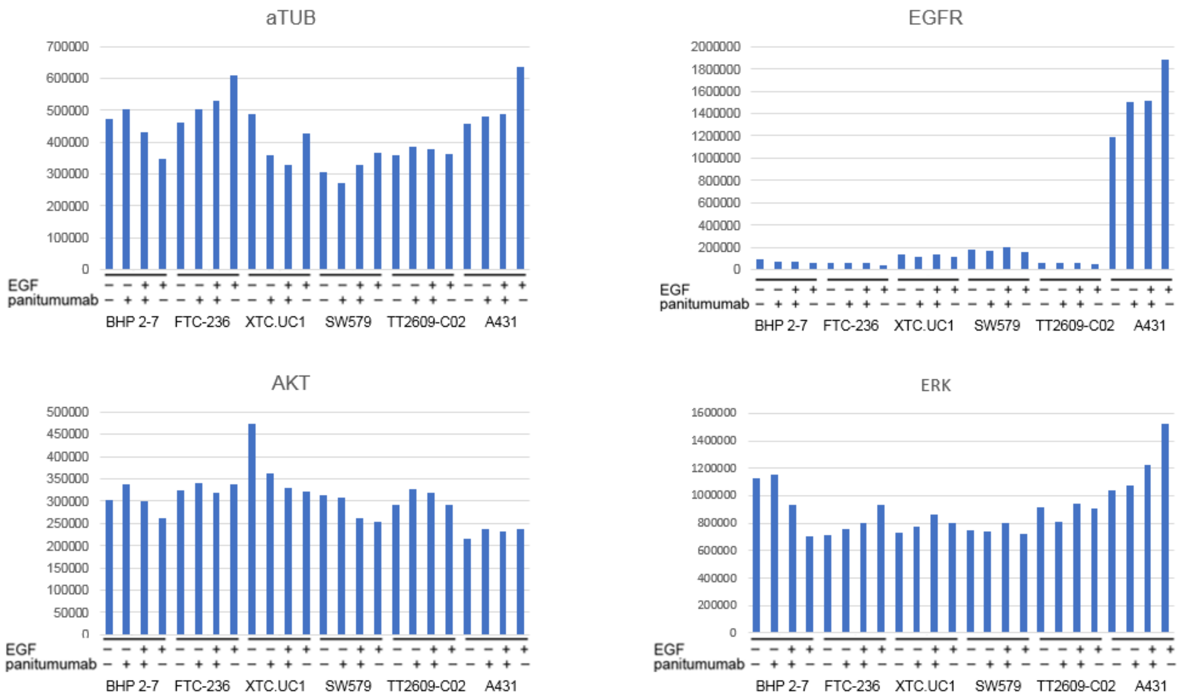
aTUB
EGFR
AKT
ERK
EGF
panitumumab
BHP 2-7
FTC-236
XTC.UC1
SW579
TT2609-C02
A431

C.

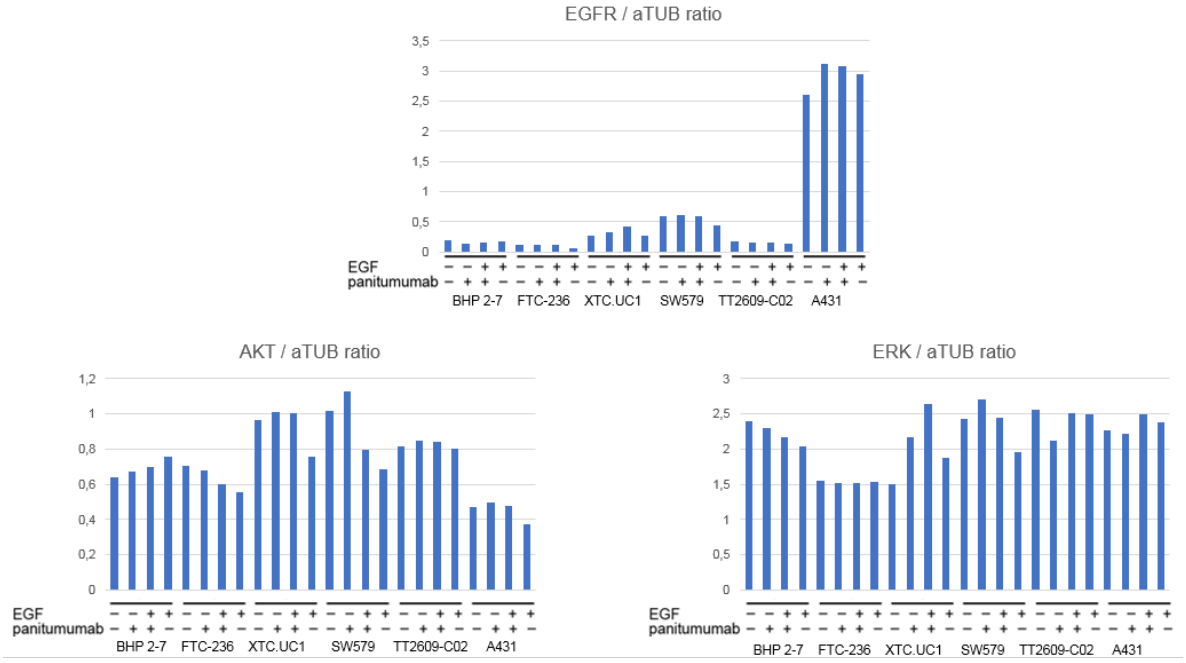
EGFR / aTUB ratio
AKT / aTUB ratio
ERK / aTUB ratio
EGF
panitumumab
BHP 2-7
FTC-236
XTC.UC1
SW579
TT2609-C02
A431

D.

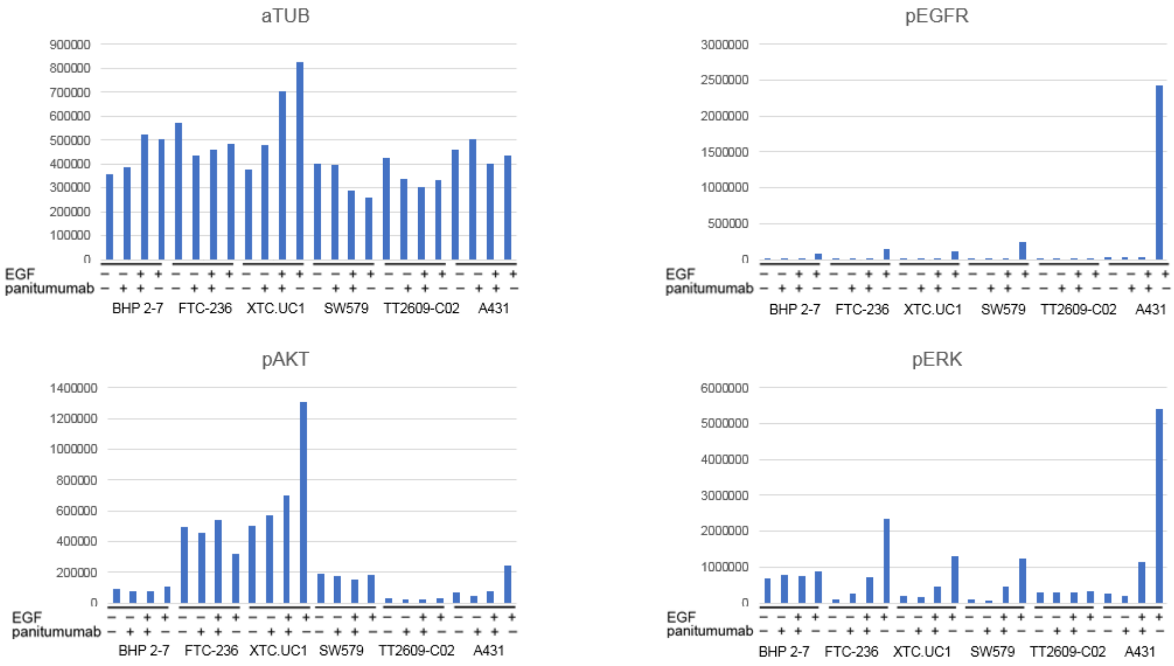

E.

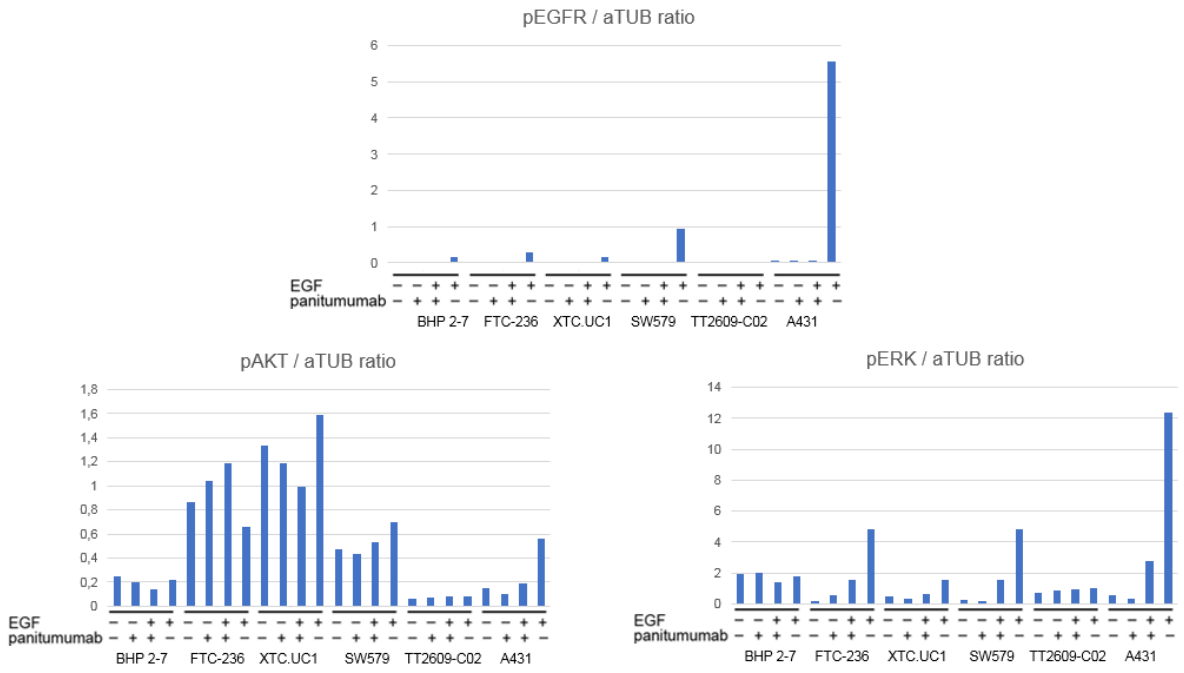

Figure S6. Quantification of intensities on the fluorescent western blot shown in Figure 5. (**A**) Quantification of fluorescent western blot intensities on raw images of blots presented in Figure 5. (Upper two blots show the expression of the proteins (EGFR, AKT, α-tubulin, ERK). The lower two blots show the phosphorylated proteins (pEGFR, pAKT, pERK, and as loading control α-tubulin). Every four lanes represent the EGF unstimulated/panitumumab untreated, unstimulated/treated, stimulated/treated, stimulated/untreated conditions per cancer cell line: BHP 2-7, FTC-236, XTC.UC1 (on the blots on the left), SW579, TT2609-C02, A431 (on the blots on the right). (**B**) Graphical presentation of measured intensities on the fluorescent western blot image for the presence of the proteins α-tubulin, EGFR, AKT, ERK and (**C**) the α-tubulin adjusted ratios of EGFR, AKT, ERK: the cancer cell line A431 shows a more than 4-fold higher EGFR expression, as compared to the thyroid cancer cell lines. (**D**) Graphical presentation of measured intensities on the fluorescent western blot image for α-tubulin, the phospho-proteins pEGFR, pAKT, pERK and (**E**) the α-tubulin

adjusted ratios of pEGFR, pAKT, pERK: pEGFR is expressed in the cancer cell lines upon EGF stimulation (every fourth lane) and potently inhibited by panitumumab (every third lane); control cell line A431 expresses >6 fold pEGFR upon EGF stimulation compared to the thyroid cancer cell lines. EGF unstimulated/panitumumab untreated pAKT levels are ~2–13 times higher in FTC-236 and ~3–20 times higher XTC.UC1 cell lines harboring a NHG compared to the other cell lines. Even with panitumumab treatment in FTC-236 and XTC.UC1 cells pAKT intensity levels remain high. pERK levels show clear response to panitumumab treatment upon EGF stimulation in cancer cell lines (compare high intensities in EGF stimulated/panitumumab untreated lane 4, to decreased intensities in EGF stimulated/panitumumab treated lane 3), except for BHP 2-7 (harboring a *RET/PTC* rearrangement) and TT2609-C02 (harboring a *NRAS* variant) which show constitutive activation of pERK.

© 2019 by the authors. Licensee MDPI, Basel, Switzerland. This article is an open access article distributed under the terms and conditions of the Creative Commons Attribution (CC BY) license (http://creativecommons.org/licenses/by/4.0/).
